# Collagen cross-linking with riboflavin and ultraviolet-A light in keratoconus: One-year results

**DOI:** 10.4103/0974-620X.48420

**Published:** 2009

**Authors:** Maria Clara Arbelaez, Maria Bernardita Sekito, Camila Vidal, Sanak Roy Choudhury

**Affiliations:** Muscat Eye Laser Center, Muscat, Oman

**Keywords:** Corneal scarring, cross-linking, irregular astigmatism, keratoconus

## Abstract

**Background:**

The aim of this study is to evaluate the safety and effectiveness of riboflavin-ultraviolet type A (UV-A) light rays induced cross-linking of corneal collagen in improving visual acuity and in stabilizing the progression of keratoconic eyes. The method of corneal cross-linking using riboflavin and UV-A light is technically simple and less invasive than all other therapies proposed for keratoconus. It is the only treatment that treats not only the refractive effects of the condition but the underlying pathophysiology.

**Materials and Methods:**

In this prospective, nonrandomized clinical study, 20 eyes of 19 patients with keratoconus were treated by combined riboflavin UV-A collagen cross linking. The eyes were saturated with riboflavin solution and were subjected for 30 min under UV-A light with a dose parameter of 3 mW/cm^2^. Safety and effectiveness of the treatment was assessed by measuring the uncorrected visual acuity, best corrected visual acuity, manifest cylinder/sphere, keratometry, pachymetry, posterior and anterior elevations from Pentacam and corneal aberrations at 6 months and 1 year post-treatment.

**Results:**

Comparative analysis of the pre-operative and 1 year post-operative evaluation showed a mean gain of 4.15 lines of UCVA (*P*= 0.001) and 1.65 lines of BCVA (*P*= 0.002). The reduction in the average keratometry reading was 1.36 D (*P*= 0.0004) and 1.4 D (*P*= 0.001) at the apex. Manifest refraction sphere showed a mean reduction of 1.26 D (*P*= 0.033) and 1.25 D (0.0003) for manifest cylinder. Topo-aberrometric analysis showed improvement in corneal symmetry.

**Conclusion:**

Cross-linking was safe and an effective therapeutical option for progressive keratoconus.

## Introduction

Keratoconus is a progressive, noninflammatory, bilateral (but usually asymmetrical) disease of the cornea, characterized by paraxial stromal thinning that leads to corneal surface distortion.[1] The thinning and the protrusion in keratoconus induces irregular astigmatism, myopia and scarring resulting in visual loss and mild to marked impairment in the quality of vision. Among the risk factors of this condition is genetics, usually inherited in an autosomal dominant fashion.[2] This could partly explain why keratoconus is a relatively common corneal disease entity in the gulf countries particularly in Oman where intermarriage with a second- or third-degree relative is a common practice.

Spectacles and contact lenses are the usual treatment modalities in the early stages of keratoconus. As the disease advances, severe corneal astigmatism and stromal opacities develop to the point where contact lenses can no longer provide useful vision and penetrating keratoplasty (PKP) becomes necessary to restore visual function. Penetrating keratoplasty is the most commonly performed surgical procedure for keratoconus, but is associated with complications including graft rejection.[3] It is estimated that eventually 21% of the keratoconus patients require surgical intervention (PKP) to restore corneal anatomy and eyesight.[4]

In selected cases wherein the cornea is still transparent and in relatively young patients who are reluctant to pursue PKP, less invasive surgical interventions may be resorted to and these are lamellar keratoplasty (LKP)[5–7] and intrastromal corneal ring segments (Intacs). LKP has the advantages of being extraocular, reversible if tissue complications occur and has the ability to replace only selected areas of diseased corneal tissue with healthy donor tissue.[8] Intacs, which were initially used to correct low myopia, have been shown to improve vision in keratoconus[9,10] and post-LASIK ectasia.[11,12]

Results of the currently available treatments for keratoconus (rigid contact lens, LKP and Intacs) are viable and are considered logical addition to the stepwise treatment of keratoconus for the improvement of vision. However, there is a new procedure that addresses primarily the pathophysiology of keratoconus and this is riboflavin UV-A rays induced cross-linking. Cross-linking of the cornea is a procedure that can increase the ties or chemical bonds between the fibers of the corneal collagen by means of a highly localized photo-polymerization using UV-A light and a photosensitizer riboflavin drops.[13,14] Riboflavin (Vitamin B2) has a dual function of acting as a photosensitizer for the production of oxygen free radicals, which induce physical cross linking of collagen, and it gives a “shielding effect” by absorbing the UV-A irradiation (90%), thereby preventing damage to deeper ocular structures. UV-A light of 370 nm wavelength at 3 mW/cm^2^ allows approximately 95% of the UV light to be absorbed into the cornea; thus there is no risk for damage to the lens and retina. Collagen cross-linking is the only treatment that deals with not only the refractive effects of the condition but the underlying pathophysiology. The aim of this study is to evaluate the safety and effectiveness of riboflavin UV-A light-induced cross-linking of corneal collagen in improving visual acuity and in stabilizing the progression of keratoconic eyes.

## Materials and Methods

This prospective longitudinal study comprised patients with signs of progressive keratoconus defined as an increase in maximum K readings in several consecutive measurements over a period of 3 to 6 months, changes in refraction, patient reports of deteriorating visual acuity and contact lens intolerance. All of the patients had bilateral keratoconus without sub-epithelial scarring, were older than 18 years old, with a corneal thickness of at least 400 µm. The eye with the more advanced stage of keratoconus was treated. The institutional ethics committee approved the study, and all patients were asked to sign an informed consent.

### 

#### Surgical technique

The surgical procedure consisted of topical anesthesia (instillation of oxybuprocaine 0.4% eye drops) and then manual epithelial abrasion of 6–8 mm using 17% ETOH for 20–40 s. This was done to ensure penetration of riboflavin in the stroma and that a high level of UV-A absorption was achieved. Riboflavin solution was repeatedly placed every 3 min for 30 min to allow sufficient saturation in the stroma. This was inspected by slit lamp examination as fluorescence within the anterior chamber. Then the cornea was irradiated with UV-A light at 365 nm with a dose parameter of 3 mW/cm^2^ for 30 min (UV-X device). During the treatment, riboflavin solution was applied every 5 min to saturate the cornea and drops of BSS every 2 min to moisten the cornea. After the treatment, the cornea was irrigated with 20-ml BSS solution and an antibiotic drop was instilled. Contact lens was placed after the treatment.

#### Postoperative regimen

Antibiotic eye drops (ofloxacin) and Pranoprofen 0.1% E/D were applied for one week until complete re-epithelializationwas achieved. After the cornea has completely healed, the contact lens is removed. Efemoline eye drops and artificial tears were applied for approximately 1 month.

#### Outcome measures and statistical analysis

Follow-up examination was done at 3, 6, and 12 months post-treatment. At each examination, uncorrected visual acuity, best corrected visual acuity, refraction, keratometry, corneal topography, pachymetry and corneal aberrations were recorded. SPSS statistical software was used for statistical analysis.

## Results

Twenty keratoconic eyes of 19 patients were included in the study. All patients completed 1 year and presented with moderate to severe keratoconus. Fourteen patients were men and 5 were women. The mean age was 24.4 years (range: 18–44 years).

Table 1 shows the pre-operative and postoperative findings in all patients. The surgery and the postoperative period were unremarkable in all patients. The epithelium re-epithelialized one week after the treatment. In the early post-operative period, all eyes had minimal anterior stromal corneal haze which resolved approximately 3 months post-operatively. After 6 months from the treatment, patients were given the option to wear contact lenses or to undergo intrastromal corneal ring surgery if necessary.

**Table 1:** Comparison between mean preoperative, six months and one year postoperative data

*Parameter*	*Pre-operative Mean±SD*	*Post-op 6 months Mean±SD*	*Post-op 1 year Mean±SD*
UCVA*	1.18 (20/320)±0.69	0.63 (20/80)±0.32	0.55 (20/70)±0.32
BCVA*	0.40 (20/50)±0.43	0.24 (20/30)±0.19	0.22 (20/30)±0.17
Manifest refraction sphere (D)	-3.84±5.10	-2.74±3.57	-2.58±3.22
Manifest refraction cylinder (D)	-4.04±1.52	-3.15±1.17	-2.79 ±1.13
K average (D)	49.93±5.02	48.68±4.61	48.57 ±4.54
K max apex (D)	51.89±7.99	50.42±8.09	50.49±8.35

UCVA-uncorrected visual acuity; BCVA- best corrected visual acuity; D = diopters; In logMAR values (Snellen acuity). SD = standard deviation

### 

#### Visual acuity

**Figure 1:**
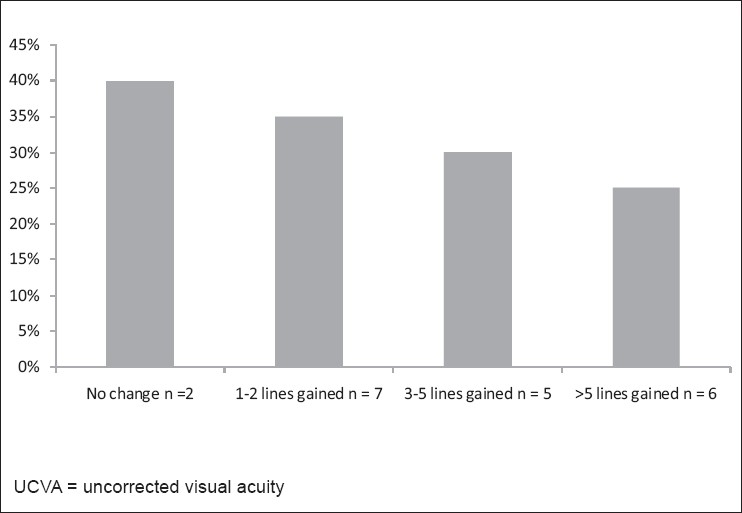
Change in UCVA from preoperative status to status 1 year following crosslinking

Visual acuity was measured using the decimal equivalent and transformed into logarithm of the minimum angle of resolution (logMAR) for further statistical analysis as recommended by Holladay.[15] Visual acuity data is expressed as logMAR ± standard deviation (Snellen value). Table 1 provides the uncorrected visual acuity (UCVA) for all patients at the pre-operative, 3 months and 6 months examination and Figure 1 shows the change in UCVA between the postoperative and one-year examinations. Two eyes maintained the preoperative UCVA; seven eyes gained one to two lines, and five eyes gained three to five lines and six eyes gained more than five lines. There was a mean gain of 4.15 lines of UCVA from preoperative to the last follow-up at 1 year.

**Figure 2:**
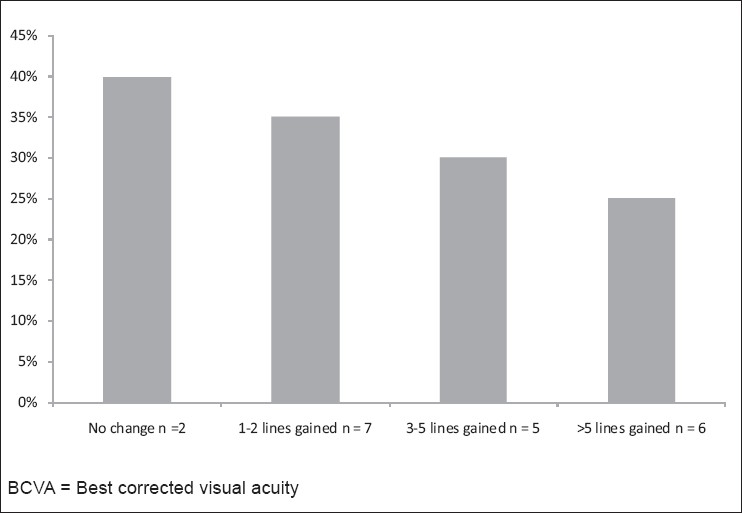
Change in BCVA from preoperative status to status 1 year following crosslinking

The best corrected visual acuity(BCVA) data from the study eyes at the pre-operative and 3 and 6 months post-operative examinations are shown in Table 1. There was a statistically significant (*P*= 0.002) improvement in BCVA between the pre-operative and 1-year evaluations. The change in BCVA lines gained or lost at 1 year compared with the pre-operative baseline is presented in Figure 2. Of the 20 eyes evaluated at 1 year, 12 of 20 eyes (60%) experienced at least a gained of 1–5 lines of BCVA. Eight of the 20 eyes (40%) experienced no change in BCVA.

#### Refractive outcome

Table 1 shows the improvement in manifest refraction sphere at the pre-operative, 6-month and 1-year evaluations. There was a statistically significant (*P*= 0.033) change in manifest refraction sphere at 1 year as compared with the pre-operative evaluation. The mean value of the manifest refraction cylinder was utilized as a measure of the change in the refractive astigmatism. The cylinder values at 1-year examination were statistically significantly less than the pre-operative measurements (*P*= 0.0003).One year after the cross-linking treatment, manifest sphere decreased by a mean of –2.75 D in 13 eyes (65%), and no improvement in 7 eyes (35%). Manifest cylinder decreased by a mean of –1.68 D in 15 eyes (75%) and no change in 5 eyes (25%).

#### Keratometry

**Figure 3:**
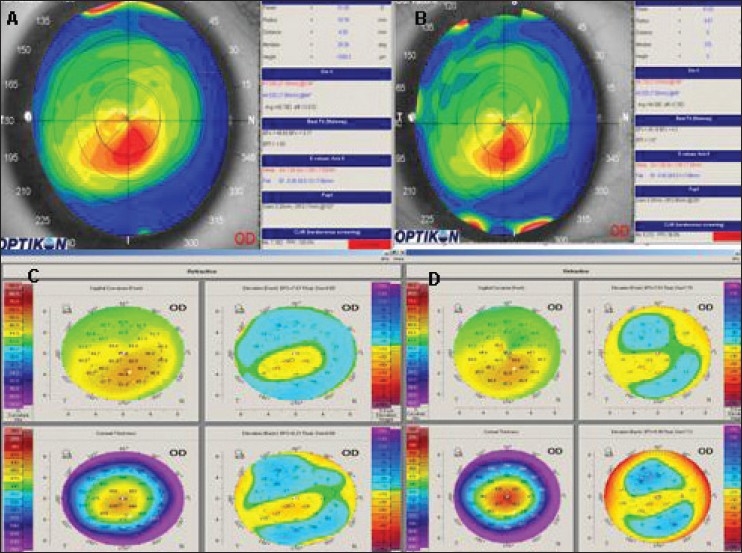
Corneal topography of a patient who had cross-linking in the right eye. A: Preoperative UCVA: –0.70 (20/100), BCVA: -0.10 (20/25), K max at the apex = 47.78. B: 1 year after cross-linking, UCVA: –0.20 (20/30), BCVA: –0.10 (20/25), K max at the apex = 45.86, K average = 44.64. C: Pentacam pre-operative, anterior elevation = +17 µm, posterior elevation = +27 µm. D: 1 year after cross-linking, reduction in anterior elevation = +4 µm and posterior elevation = +17 µm.

The K value at the apex decreased by a mean of 1.40 D from pre-operative to 1-year evaluation, *P* = 0.01.The K average decreased by a mean of 1.36 D from pre-operative to 1-year evaluation, *P* =0.004. Table 1 and Figure 3 describes the change in K average and K value at the apex from pre-operative value to 1 year.

#### Pachymetry

**Figure 4:**
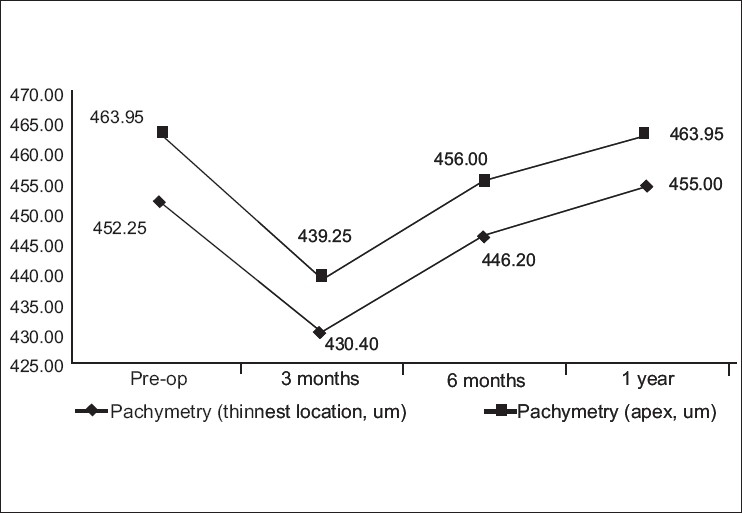
Changes in pachymetry measurements (µm) at the thinnest location and at the apex

Pachymetry measurements (measured by the Pentacam) at the thinnest location and at the apex were measured pre-operatively, 3-months, 6-months and 1-year post-operatively. At 3-months post-operative examination, there was a significant reduction in pachymetry both at the thinnest location (*P*= 0.0007) and at the apex (*P*= 0.0002). Pachymetry at the thinnest location reduced from 452.25 ±29.58 µm pre-operatively to 430.4 ±44.38 µm at 3 months (4.83% reduction). At the apex, there was also a significant decline from 463.96 ±27.28 µm pre-operatively to 439.25 ±42.80 µm at 3 months (5.32% reduction). One-year evaluation showed the pachymetry to increase gradually to 455 ±37.98 at the thinnest location and 463.95 ±37.36 at the apex. Figure 4 shows the changes in pachymetry measurement at the thinnest location and at the apex in time.

#### Corneal wavefront surface aberrometry

**Figure 5:**
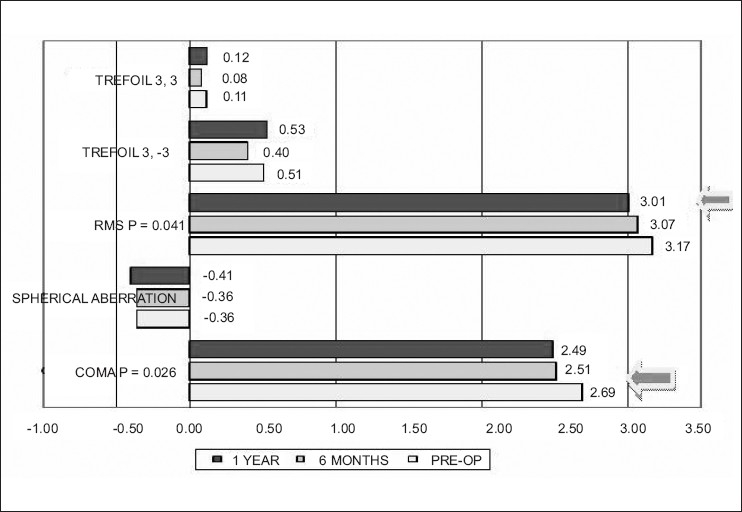
Corneal wavefront analysis with 4-mm pupil; blue arrows indicate (paired t tests) signifi cant difference with preoperative data

Corneal wavefront surface aberommetry showed a significant reduction in absolute RMS (*P*= 0.041) and absolute coma (*P*= 0.026) at 1 year with respect to the pre-operative value [Figure 5]. Spherical and other high-order aberrations did not show any significant change.

#### Anterior and posterior elevation

Anterior elevation at the thinnest location and at the apex were measured pre-operatively, 6-months and at 1-year post-treatment by the Oculus Pentacam. There was a significant reduction in anterior elevation both at the thinnest location and at the apex at 6 months post-treatment. At the thinnest location, the anterior elevation decreased significantly, *P*= 0.015, from 31.25 ± 17.06 D pre-operatively to 26.35 ±16.63 D at 6 months post-treatment. No significant change was noted at 1 year post-treatment. At the apex, the anterior elevation decreased significantly, *P*= 0.025, from 21.05 ±15.55 pre-operatively to 17.0 ±15.37 D at 6 months post-treatment. No significant change was noted at 1 year post-treatment.

Student’s *t* test for paired data did not find any significant difference in the posterior eleavation at the thinnest location and at the apex from pre-operative value, at 6 months and at 1 year post-treatment. Table 2 and Figure 3 present the changes in anterior and posterior elevation at the thinnest location and at the apex over time.

**Table 2:** Anterior surface and posterior surface elevation change at the thinnest location and at the apex from pre-operative, 6 months and one year post evaluation as measured by the 0cuius Pentacam

	*Pre-op*	*6 months*	*1 year*
Anterior elevation, thinnest location (D)	31.25 ± 17.06	26.35 ± 16.63	28.45 ± 20.97
Anterior elevation, apex (D)	21.05 ± 15.55	17 ±15.37	18.8 ± 15.33
Posterior elevation, thinnest location (D)	54.35 ± 29.98	49.95 ± 28.87	50.45 ± 30.45
Posterior elevation, apex (D)	30.45 ± 25.12	30.55 ± 23.96	31.3 ± 23.77

## Discussion

The goal for the corneal collagen cross-linking treatment is to delay or halt the progression of keratoconus and to defer the need for a corneal transplant. The results of this study were encouraging as far as safety and effectiveness are concerned. No side effects were noted except for the subjective complaints of patients, namely, visual symptoms like fluctuating vision and double images. Although no survey was used in the study, patients anecdotally reported improvement in visual symptoms over time.

Refractive results in this study were approximately similar to other studies published.[16,17,18] There was a 1.25-D reduction in the manifest sphere and cylinder as confirmed by the reduction in the keratometry readings. This reduction in refractive error is also associated with a significant increase in UCVA (4 Snellen lines).

Corneal wavefront surface aberrometric analysis reflected a significant reduction in RMS and comatic aberrations. This could partly explain the improvement in the BCVA in 60% of the patients.

In a study made by Wollensak *et al*,[19] it was shown that apoptotic cell death occurs after exposure to UV-A light. The massive, transient cellular damage or keratocyte apoptosis is assumed to be an initiator of the corneal wound healing response and the start of the complex wound healing cascade.[20] In the present study, a 5% reduction in pachymetry was observed in all patients at 3 months. After which, a steady increase was noted. This finding could correspond to the apoptosis that occurs after the treatment (2 to 3 months) and the repopulation that occurs thereafter (6 months). Based on this finding, the authors strongly suggest that when the cross-linking treatment is combined with an additional treatment such as Intacs or LASEK, a healing interval of approximately 2 to 3 months should be respected to avoid complications caused by the additional damage of the added procedure.

In the present study, a significant reduction in the anterior elevation was noted but the reduction in posterior elevation was not statistically significant. The studies in animal experiments[21,22] and in humans[23,24] may provide an insight to this finding. These studies have shown that treatment of the cornea with riboflavin and UV-A significantly stiffened the cornea only in the anterior 300 µm. This depth dependent stiffening effect may explain significant flattening in the anterior cornea as revealed by the reduction in the anterior elevation.

It has been shown that collagen cross-linking increases the biomechanical rigidity of the cornea by 4.5 times.[21] By increasing the biomechanical stability of the cornea using the riboflavin and UV-A-induced collagen cross-linking, it is possible to stop the progression of keratoconus. The improvement in vision, reduction in the refractive effect, reduction in keratometry readings, improvement in the topographic and surface aberrometric analysis are all evidences that the treatment can arrest the progression of keratoconus. No analysis of the fellow eye was done in this study; such analysis is indicated in the future.

Given the effectiveness, simplicity, safety and cost effectiveness (this is a one-time treatment) of this modality, corneal collagen cross linking has the potential to become a standard therapy for progressive keratoconus in the future. Particularly in Oman, this treatment could benefit a lot of people due to the fact that there are very few centers that are capable of performing corneal transplant and the environment is not suitable for contact lens wear. However, as with all new treatment modalities, controversies and questions remain unanswered. Long-term results are necessary to evaluate the duration of the stiffening effect, indications and contraindications must be investigated, hence, the need for long term longitudinal studies.
